# Nature of the Dirac gap modulation and surface magnetic interaction in axion antiferromagnetic topological insulator ${\hbox {MnBi}}_2 {\hbox {Te}}_4$

**DOI:** 10.1038/s41598-020-70089-9

**Published:** 2020-08-06

**Authors:** A. M. Shikin, D. A. Estyunin, I. I. Klimovskikh, S. O. Filnov, E. F. Schwier, S. Kumar, K. Miyamoto, T. Okuda, A. Kimura, K. Kuroda, K. Yaji, S. Shin, Y. Takeda, Y. Saitoh, Z. S. Aliev, N. T. Mamedov, I. R. Amiraslanov, M. B. Babanly, M. M. Otrokov, S. V. Eremeev, E. V. Chulkov

**Affiliations:** 1 Saint Petersburg State University, 198504 Saint Petersburg, Russia; 2 Hiroshima Synchrotron Radiation Center, Hiroshima University, Hiroshima, Japan; 3 Department of Physical Sciences, Graduate School of Science, Hiroshima University, Hiroshima, Japan; 4 ISSP, University of Tokyo, Kashiwa, Chiba 277-8581 Japan; 5 Materials Sciences Research Center, Japan Atomic Energy Agency, Sayo, Hyogo 679-5148 Japan; 6 Azerbaijan State Oil and Industry University, AZ1010 Baku, Azerbaijan; 7 Institute of Physics, ANAS, AZ1143 Baku, Azerbaijan; 8 Baku State University, AZ1148 Baku, Azerbaijan; 9 Institute of Catalysis and Inorganic Chemistry, ANAS, AZ1143 Baku, Azerbaijan; 10 Centro de Física de Materiales (CFM-MPC), Centro Mixto CSIC-UPV/EHU, 20018 Donostia-San Sebastián, Basque Country Spain; 11 IKERBASQUE, Basque Foundation for Science, 48011 Bilbao, Basque Country Spain; 12 Institute of Strength Physics and Materials Science, 634055 Tomsk, Russia; 13 Tomsk State University, 634050 Tomsk, Russia; 14 Donostia International Physics Center (DIPC), 20018 Donostia-San Sebastián, Basque Country Spain; 15 Departamento de Física de Materiales, Facultad de Ciencias Químicas, UPV/EHU, Apdo. 1072, 20080 San Sebastián, Spain

**Keywords:** Condensed-matter physics, Quantum Hall, Surfaces, interfaces and thin films, Topological matter, Electronic properties and materials, Magnetic properties and materials, Materials science, Physics

## Abstract

Modification of the gap at the Dirac point (DP) in axion antiferromagnetic topological insulator ${\hbox {MnBi}}_2 {\hbox {Te}}_4$ and its electronic and spin structure have been studied by angle- and spin-resolved photoemission spectroscopy (ARPES) under laser excitation at various temperatures (9–35 K), light polarizations and photon energies. We have distinguished both large (60–70 meV) and reduced ($<20~ \hbox {meV}$) gaps at the DP in the ARPES dispersions, which remain open above the Neél temperature ($T_{\mathrm{N}} = 24.5~ \hbox {K}$). We propose that the gap above $T_{\mathrm{N}}$ remains open due to a short-range magnetic field generated by chiral spin fluctuations. Spin-resolved ARPES, XMCD and circular dichroism ARPES measurements show a surface ferromagnetic ordering for the “large gap” sample and apparently significantly reduced effective magnetic moment for the “reduced gap” sample. These observations can be explained by a shift of the Dirac cone (DC) state localization towards the second Mn layer due to structural disturbance and surface relaxation effects, where DC state is influenced by compensated opposite magnetic moments. As we have shown by means of ab-initio calculations surface structural modification can result in a significant modulation of the DP gap.

## Introduction

Interplay between topology and magnetism plays a significant role in generation of a number of exotic topological quantum effects such as Quantum Anomalous Hall Effect (QAHE)^1–4^ and topological magnetoelectric effect^1, 5, 6^. These effects, discovered in magnetic topological insulators (TIs), are very important for both fundamental science and future technological applications, like dissipation-less topological electronics and topological quantum computation. They are accompanied by a predicted opening of a gap at the Dirac point (DP) arising as a result of the time reversal symmetry (TRS) breaking due to the induced magnetic ordering. In turn, this magnetic gap and its magnitude can be good indicators of the developed effects and their modification under different conditions. Intrinsic magnetic TIs (see, for example, Refs.^7–18^) are currently viewed as the best candidates for implementing the aforementioned effects. Thus, these compounds can be considered as a very promising platform for realizing other interesting and exotic effects, such as magnetic monopole and axion field and their possible manipulation^1, 19–22^.

Recently, an intrinsic antiferromagnetic (AFM) TI with ${\hbox {MnBi}}_2 {\hbox {Te}}_4$ stoichiometry has been successfully synthesized, and its electronic structure has been theoretically and experimentally investigated^7–13^. This compound has layered crystal structure consisting of septuple layers (SLs) with van der Waals (vdW) bonding between them. Each SL contains Mn layer in the central plain with ferromagnetic coupling between Mn magnetic moments. An overall AFM ordering in the compound is formed by the nearest neighboring Mn FM layers coupled antiferromagnetically with each other. As a result, all magnetic moments are ordered and aligned with the *c*-axis, i.e. perpendicular to the surface (in the out-of-plane direction). The topological surface state is formed at ${\hbox {MnBi}}_2 {\hbox {Te}}_4$(0001) due to the inverted Bi $p_z$ and Te $p_z$ bulk bands at the $\Gamma$-point due to strong spin-orbit coupling, which is essentially the same as for ${\hbox {Bi}}_2 {\hbox {Te}}_3$^7, 9–11^. Despite the fact that due to AFM coupling, TRS ($\Theta$) is broken, there are two symmetries: $\mathrm {P}_2\Theta$ and $\mathrm {S} = \Theta \mathrm {T}_{1/2}$ (where $\mathrm {P}_2$ is the inversion operation centered between neighboring SLs, and $\mathrm {T}_{1/2}$ is a lattice translation), which preserve the topological invariant, and allow this compound to be AFM TI. Theoretical calculations predict for AFM TI ${\hbox {MnBi}}_2 {\hbox {Te}}_4$ a magnetically-driven energy gap at the DP of 88 meV^7^.

Experimentally measured DP gap varies from 50 to 85 meV depending on photon energy and the measurements conditions, see, for instance, Refs.^7, 11, 12, 23^. Moreover, it was found that the Dirac cone (DC) state remain practically temperature independent above and below the Neél temperature ($T_{\mathrm{N}}$), although certain temperature dependence of the photoemission intensity and lineshape was also observed^7^. Similar results, which show the gapped DC state at temperature above $T_{\mathrm{N}}$ were also reported for the Gd-doped TI^17, 24^ and other kinds of magnetically-doped TIs (see, for instance, Refs.^6, 25–29^). At the same time, a series of works^14–16, 30^ has appeared in literature where a “gapless”-like ARPES dispersion was observed for ${\hbox {MnBi}}_2 {\hbox {Te}}_4$. However, for these measurements a small DP gap of about 13 meV can be also distinguished, which also remains open above $T_{\mathrm{N}}$^14, 30^. The reduced gap for these samples has been attributed to the difference between the bulk and surface magnetic orders, although it should be noted that only a weak temperature dependence of the spectra has been revealed. The significant difference in the gap width observed for different samples of ${\hbox {MnBi}}_2 {\hbox {Te}}_4$ and its weak dependence on the long-range magnetic order transition have not been satisfactorily explained so far.

In the present work we have carried out a detailed analysis of the DP gap in ${\hbox {MnBi}}_2 {\hbox {Te}}_4$ based on the Spin and Angle- resolved Photoemission Spectroscopy (spin-/ARPES) measurements performed both above and below $T_{\mathrm{N}}$ (24.5 K) with variation of the photon energy and polarization. We show that DP gap of this compound is slightly reduced, but remains open above $T_{\mathrm{N}}$. We attribute it to the chiral-like spin fluctuations which can be considered as a local emergent magnetic field preserving the gap. We present and compare the results of the ARPES measurement for different kinds of the ${\hbox {MnBi}}_2 {\hbox {Te}}_4$ samples (or different surface areas for one sample), demonstrating a large (60–70 $\hbox {meV}$) and reduced ($<20~ \hbox {meV}$) gap at the DP. Both kinds of the samples are characterized by the same perfect X-ray diffraction^13^. We have studied these samples by XMCD, CD and spin-resolved ARPES and have shown the possibility of differences in the formed surface magnetic moment, which affects the DC state differently, for the samples with different gap at the DP. These effects can be related to a shift of the topological DC state towards the second SL block, due to surface structural modification, where it senses simultaneously the opposite magnetic moments of the first and the second Mn layers, as it is revealed by our ab-initio simulations. Thus, this shift leads to a decrease in the effective magnetic moment, which acts on the DC state.

## Results

### ARPES dispersion maps

**Figure 1:**
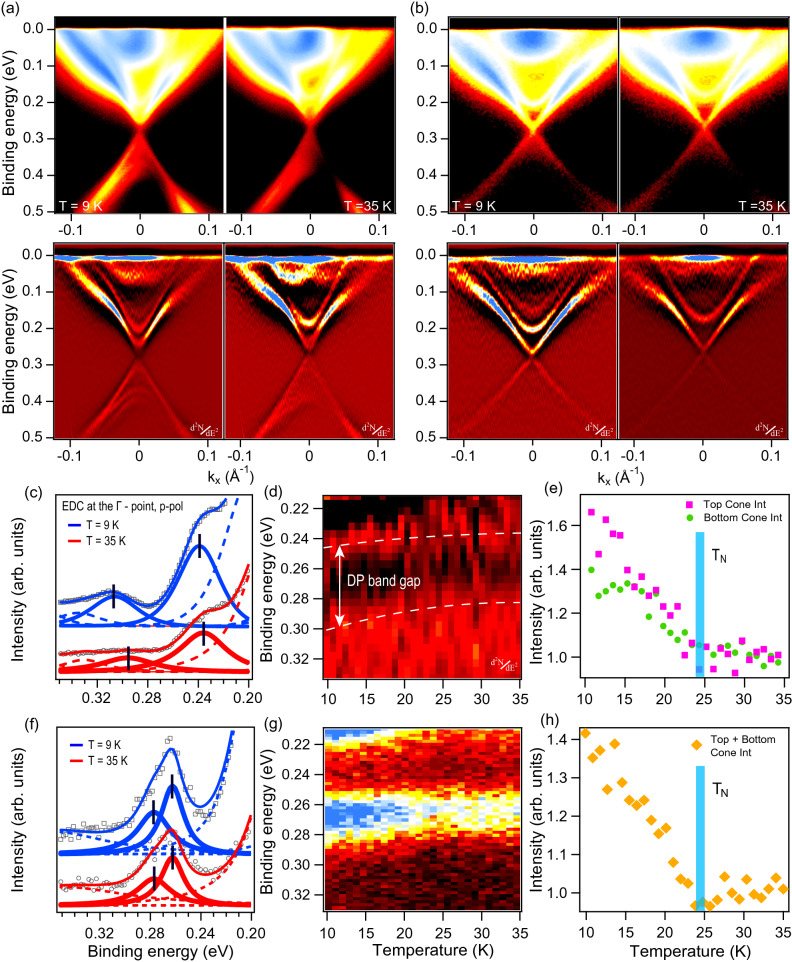
(**a**,**b**) upper line—ARPES dispersion maps measured for ${\hbox {MnBi}}_2 {\hbox {Te}}_4$ at photon energy 6.3 eV using p-polarized laser radiation below (9 K) and above $T_{\mathrm{N}}$ (35 K) for the sample with a large (**a**) and reduced gap (**b**). (**a**,**b**) lower line—the same as in the upper line but in the $d^2N/dE^2$ representation. (**c**) EDCs cut at the $\Gamma$-point ($k_\parallel =0~\AA ^{-1}$) in energy region around the DP for the “large gap” sample at 9 K (blue curves) and 35 K (red curves) with corresponding spectral decompositions. The modification of the DC structure near the DP under gradual increase of temperature between 9 and 35 K shown as a set of EDCs at the $\Gamma$-point (**d**) and as integrated intensity of corresponding EDCs in the energy intervals corresponding to the upper (pink) and lower (green) DC parts (**e**). (**f**–**h**) The same as in panels (**c**–**e**) measured for sample with a “reduced gap”. Note that no distinction between the upper and lower parts of the DC is made in (**h**).

Figure 1a,b-upper row show a comparison between the ARPES dispersion maps measured for ${\hbox {MnBi}}_2 {\hbox {Te}}_4$ below and above $T_{\mathrm{N}} = 24.5~ \hbox {K}$^7, 13^ for samples or surface areas which hereafter we will call as sample with a “large gap” at the DP (a) and sample with “reduced gap” at the DP (b), see details in the text below. In the bottom row in panels (a, b) the corresponding ARPES maps in the $d^2N/dE^2$ form are shown for better visualization. For the measurements presented in panels (a) and (b) we used the $\mu$-Laser ARPES system ($h\nu =6.3~\hbox {eV}$) at the Hiroshima Synchrotron Radiation Center^31^ with improved angle, energy and spatial resolutions. From the dispersion maps at 9 K (a,b) the positions of the edges of the conduction and valence bands (CB and VB) are located at about 0.22 eV and 0.36 eV binding energies (BEs), respectively. At temperature of 35 K the CB and VB edges respectively shift to BEs of approximately 0.19 and 0.38 eV. This difference is due to the exchange splitting of the bulk states below $T_{\mathrm{N}}$ leading to decrease of a total fundamental gap (see Ref. ^23^ for more details and analysis of these states and their splitting).

One can see from ARPES dispersion in panel (a) that the energy gap at the DP seems to be open both below and above $T_{\mathrm{N}}$. For more quantitative estimation in Fig. 1c we present spectral decomposition of Energy Distribution Curves (EDCs) at the $\Gamma$-point ($k_\parallel =0~\AA ^{-1}$) at $T=9~ \hbox {K}$ (blue curves) and 35 K (red curves). A significant dip in photoemission intensity is seen at 9 K between upper and lower parts of the DC (marked by black lines) and it remains visible even above $T_{\mathrm{N}}$. The fitting yields value of the DP gap $\sim 70~ \hbox {meV}$ at 9 K that slightly decreases to 60 meV at 35 K. Considering the full width at half maximum of the spectral components the error in the estimations is about 20 meV. Although the estimation above $T_{\mathrm{N}}$ becomes somewhat less accurate due to reduced intensity and sharpness of the DC components, such reduction can be treated as important evidence of the magnetism influence on the electronic structure (Refs. ^7, 23^).

To investigate the evolution of the DP gap with temperature, we performed a set of measurements between 9 and 35 K. In Fig. 1d, the variation of the DC components intensity is presented, where the white dashed lines approximately show the positions of their maxima. The DC components are located at BEs of about 0.245 eV and 0.3 eV at 9 K and they gradually shift towards lower BEs while the temperature increases. As one can see, the DP gap magnitude decreases continuously up to $T_{\mathrm{N}}$, but remains almost constant above it. Additionally, Fig. 1e demonstrates variation of the spectral weight of the DC components with temperature (separately for its upper and lower parts). One can clearly see that the intensities gradually decrease with temperature up to $T_{\mathrm{N}}$ after which they remain at an approximately constant level. Thus, the presented results confirm the opening of a large DP gap which has slight temperature variation and is mitigated above $T_{\mathrm{N}}$.

Besides, we observed samples of another type that, although being measured at the same conditions, demonstrate significantly reduced DP gap. Figure 1b shows the ARPES dispersion maps for this kind of samples, measured at temperatures below and above $T_{\mathrm{N}}$. A dataset obtained with the same treatment as for discussed above “large gap” sample is shown in panels (f-h) for the sample with “reduced gap”. Spectral decomposition of EDCs (panel (f)) yields the DP gap size less than 20 meV, independently on temperature, which is consistent with the report of Ref. ^30^. Figure 1g directly demonstrates the behavior of the DC components near the DP upon crossing $T_{\mathrm{N}}$. All visible variations of the line width are related to the intensity decrease. Integrated intensity of the DC components (panel (h)) behaves qualitatively similar to the one observed for the sample with the “large gap” (panel (e)). Moreover, the exchange splitting of the CB state (at $\sim 0.2~ \hbox {eV BE}$^23^) for both samples below $T_{\mathrm{N}}$ has almost the same value (see $d^2N/dE^2$ in (a) and (b)). Thus, one can expect the identity of the bulk magnetic ordering for both samples which though demonstrate significantly different DP gap values.

### Resonant photoemission measurements

In Refs. ^28, 32, 33^ it was proposed that the DP gap in magnetically-doped TIs can have a non-magnetic origin and appears due to the avoided crossing hybridization of the DC with the impurity levels of magnetic atoms near the DP. Thus, to confirm possible magnetic-derived origin of the gap opening (i.e. absence of Mn-levels near the DP) we performed resonant PE measurements. This method allows one to enhance the contribution of the Mn-derived states in PE spectra and to indicate the exact energy range of localization of these states.

**Figure 2:**
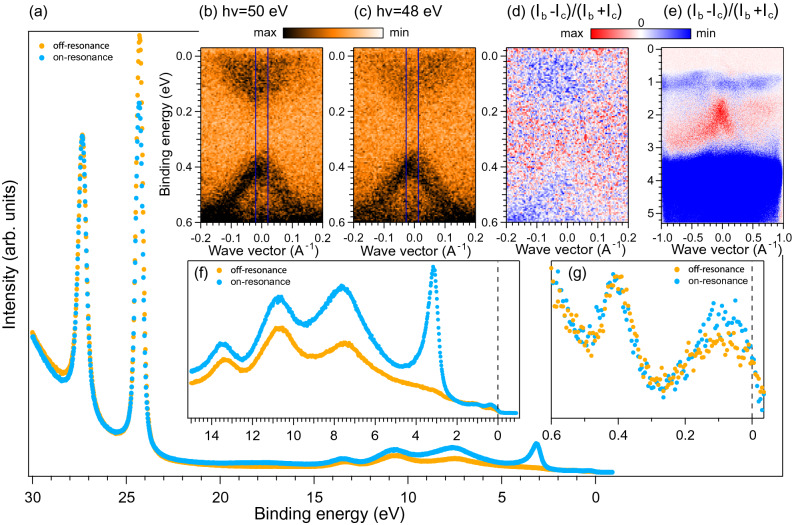
(**a**) Resonant Mn($3p-3d$) PE spectra measured at on-resonance energy $h\nu =50~ \hbox {eV}$ (blue curve) and off-resonance energy $h\nu =48~ \hbox {eV}$ (orange curve). A zoom into the region up to the BE of 15 eV is shown in (**f**). (**b**,**c**) On- and off-resonance ARPES dispersion maps measured in the region of the DC with their difference shown in (**d**). (**e**) The same as in panel (**d**), but up to higher BEs. (**g**) On- and off-resonance spectra in the DC region taken as EDCs in the vicinity of the $\Gamma$-point cut from panels (**b**) and (**c**).

According to theoretical calculations^7, 9, 34^ the Mn 3*d* states in ${\hbox {MnBi}}_2 {\hbox {Te}}_4$ are energetically separated from the topological surface state. Experimentally it is shown in Fig. 2 by resonant PE measurements at energies of Mn($3p-3d$) adsorption edge ($h\nu$ = 48–50 $\hbox {eV}$). One can clearly see a resonant increase of the Mn 3*d* states at about 3.2 eV BE (see panels (a), (e) and (f)) while there is no visible intensity change in the DC region (see panels (b–d) and (g)). This proves the absence of any notable contribution of the Mn d states in the vicinity of the DP and shows the unlikeliness of an avoided-crossing-scenario in ${\hbox {MnBi}}_2 {\hbox {Te}}_4$. This is clearly opposed to observed for Mn-doped ${\hbox {Bi}}_2 {\hbox {Te}}_3$^35^.

Panels (e,g) show some weak intensity growth on-resonance in the VB region with 1 eV BE and in the CB region that points to presence of some Mn-derived states there. They might be related to the formation of the Mn(*d*)-Te(*p*) hybrid states^34^. However, these states are unlikely to have significant contribution to the DP gap opening via the avoided-crossing hybridization mechanism. All measured samples demonstrate analogous behavior during the resonant photoemission experiment with a small variation of the intensity of the Mn-derived peak at 3.2 eV BE.

### In-plane and out-of-plane spin texture of the gapped Dirac state

**Figure 3:**
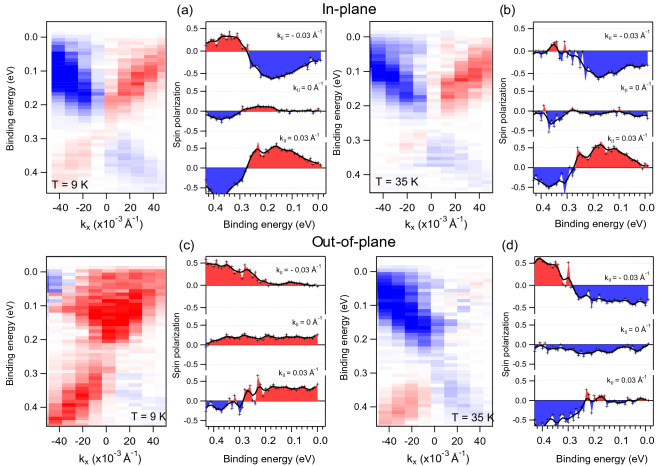
(**a**,**b**) Left parts: in-plane spin-resolved ARPES dispersion maps measured for ${\hbox {MnBi}}_2 {\hbox {Te}}_4$ along the $\Gamma \mathrm {M}$ direction using s-polarized LR ($h\nu = 7~ \hbox {eV}$) at a temperature of 9 and 35 K, respectively. (**a**,**b**) Right parts: the corresponding in-plane polarizations measured at the $\Gamma$-point and at $k = \pm 0.03~\AA ^{-1}$. (**c**,**d**) The same as in panels (**a**,**b**) but for the out-of-plane spin-resolved ARPES dispersion maps and corresponding out-of-plane polarization.

To analyze the spin structure of the DC state formed in ${\hbox {MnBi}}_2 {\hbox {Te}}_4$ we measured spin-resolved ARPES dispersion maps for the in-plane and out-of-plane spin components at temperatures both below and above $T_{\mathrm{N}}$, which are presented in Fig. 3. One can see that for the in-plane spin component (Fig. 3a,b) a helical spin structure is observed with a pronounced spin inversion for opposite branches of the DC for all measured temperatures. At the same time, for the out-of-plane spin polarization the spin-resolved dispersion maps (Fig. 3c,d) demonstrate no visible spin inversion between the upper and lower DCs. Although, for positive and negative $k_\parallel$ the magnitude of the spin polarization is sharply changed in the BE region between 0.2 and 0.3 eV. This change occurs in the background of the constant-like contribution to spin polarization which may be related to the PE-induced spin polarization of the CB states hybridized with the Mn 3*d* states. This behavior of the out-of-plane polarization is observed both below and above $T_{\mathrm{N}}$ with some change in the polarization level with temperature. The observation of the out-of-plane spin polarization above $T_{\mathrm{N}}$ is consistent with the assumption about the occurrence of spin fluctuations, as in Ref. ^7^, with a common spin texture, similar to that in Ref. ^36^, which includes both in-plane and out-of-plane spin components. Besides, the spin polarization of the Mn 3d states in the CB region (see Fig. 2) also may have a contribution to this spin-polarized background in the measured spin-ARPES dispersion maps.

Overall, the spin structure that is shown in Fig. 3 confirms that ${\hbox {MnBi}}_2 {\hbox {Te}}_4$ is characterized by the helical spin texture for temperature below and above the $T_{\mathrm{N}}$. At the same time, the measured out-of-plane spin-resolved spectra show complex structure, possibly due to the PE final state effect (as in Ref. ^27^) and the impact of the Mn 3*d* states.

### Photon energy dependence of the out-of-plane polarization of the DC state

**Figure 4:**
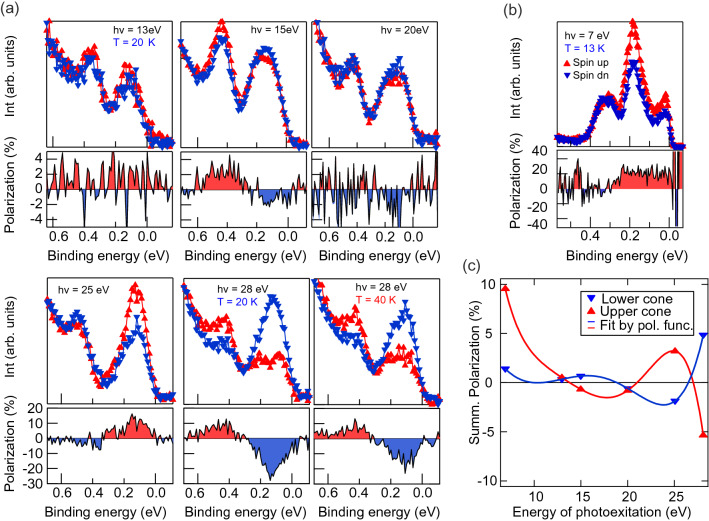
(**a**) Photon energy dependence ($h\nu =13-28~ \hbox {eV}$, SR) of the out-of-plane spin resolved spectra measured at $k_\parallel =0~\AA ^{-1}$ below (20 K) and above (40 K) $T_{\mathrm{N}}$. Upper parts of each panel show the spin-up and spin-down components, while the lower parts demonstrate corresponding out-of-plane polarization. (**b**) The same as in (**a**) but acquired with at $h\nu = 7.0~ \hbox {eV}$ (LR) and $T = 13~ \hbox {K}$. (**c**) Photon energy dependent variation of the out-of-plane polarization of the upper and lower DC state.

In order to study the out-of-plane spin polarization inversion between the upper and lower parts of the DC in the vicinity of the $\Gamma$-point and its possible variation on the photon energy we measured the photon energy dependence of the out-of-plane spin component in the spin-resolved PE spectra under photoexcitation by SR (Fig. 4a) and LR (Fig. 4b). In panel (a) one can see that spectra measured at photon energies of 15, 25 and 28 eV show inversion of the spin polarization for the upper and lower parts of the DC which confirms hedgehog-like spin structure of the DC at the $\Gamma$-point, see, for instance^25^. Moreover, one can see that the spectrum measured with $h\nu = 28~ \hbox {eV}$ and $T = 40~ \hbox {K}$ also shows an evidence of inversion of the spin polarization at the $\Gamma$-point. This also supports our assumption of the magnetic gap opening at temperatures higher than $T_{\mathrm{N}}$. Furthermore, the dependence of the polarization on the photon energy has an oscillating character. The polarization changes its sign between 25 and 28 eV (i.e. the BEs of the Bi $5d_{3/2}$ and $5d_{5/2}$ levels) and then oscillates with photon energy in magnitude and sign. Therefore, the variation of the out-of-plane polarization with photon energy has rather oscillating character (Fig. 4c) which is consistent with calculations shown in Refs. ^26, 27, 37, 38^.

At the same time, one can see that the spin-resolved spectrum measured at $h\nu =7.0~ \hbox {eV}$ (panel (b)) demonstrates an additional out-of-plane polarization contribution in the region of the CB states, almost independent on the BE. We relate it to the out-of-plane polarization of the CB states hybridized with the Mn 3*d* states^34^ which become more pronounced at the photon energies between 6 and 15 eV. Their enhancement is well visible in Fig. 2f.

Thus, the presented spin-resolved spectra demonstrate a pronounced inversion of the out-of-plane spin polarization between the upper and lower parts of the DC at the $\Gamma$-point. The value and the sign of the spin polarization oscillate with photon energy in PE spectra due to the final state effects. The observed out-of-plane spin polarization confirms a magnetic-derived origin of the DP gap. The spin-resolved spectra taken at $h\nu =28~ \hbox {eV}$ and $T = 20$ and 40 K demonstrate a slight decrease in the DC spin polarization above $T_{\mathrm{N}}$.

However, in the presented measurements performed using the SR we did not distinguish between the samples (or surface areas) characterized by large or reduced gap. In order to estimate the difference in the DC spin structure of the samples with the “large gap” and the “reduced gap” we used circular dichroism (CD) ARPES method at $\mu$-ARPES Laser station where these two kinds of samples were characterized previously (Fig. 1). CD ARPES is a useful technique to obtain information about variation of the total orbital momentum of states which in case of the TI’s DC may reflect the DC spin texture.

### Large and small gaps at different polarizations of LR and temperature

**Figure 5:**
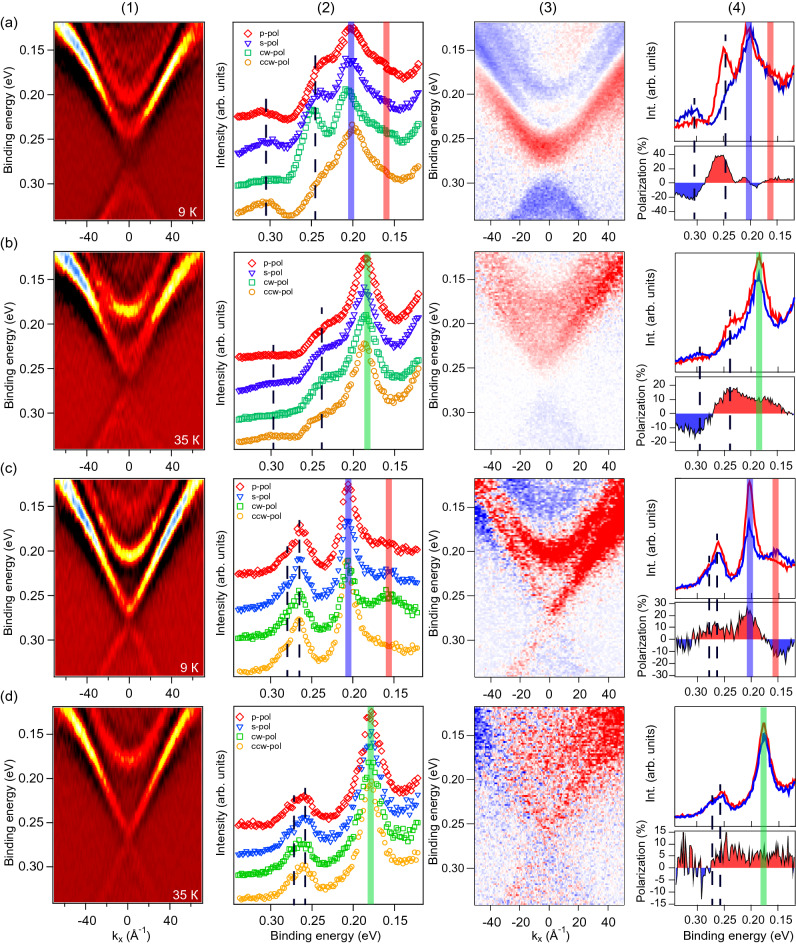
(**a1**–**d1**) ARPES dispersion maps measured for the cases of a large (**a1**,**b1**) and reduced (**c1**,**d1**) gap at the DP at temperatures of 9 K (**a1**,**c1**) and 35 K (**b1**,**d1**) using p-polarized LR (with $h\nu =6.3~ \hbox {eV}$). The spectra are shown in the region close to the DP in the $d^2N/dE^2$ presentation for better visualization of the DP gap. (**a2**–**d2**) the corresponding EDCs measured at the DP at $k_\parallel =0~\AA ^{-1}$ using different polarization of LR at temperature 9 and 35 K, respectively. CW and CCW denote circularly polarized laser radiation with clockwise and counter clockwise polarizations. (**a3**–**d3**) The CD ARPES dispersion maps obtained by subtraction of the PE signal of opposite circular polarization. (**a4**–**d4**) A comparison between the corresponding CD EDCs in the region close to the DP (upper panels) with presentation of the subtracted PE signal measured at opposite circular polarizations (bottom panels).

In Fig. 5 we compare the ARPES dispersion maps for samples with large (panels a,b) and reduced (panels c,d) gap, measured at different temperatures and polarizations of LR. In Fig. 5a1–d1 the ARPES dispersion maps, measured using *p*-polarized LR at $h\nu =6.3~ \hbox {eV}$ are presented in the $d^2N/dE^2$ form. Fig. 5a2–d2 show the corresponding EDCs measured at the $\Gamma$-point at different polarizations of LR and temperatures of 9 and 35 K. The maxima of the peaks’ intensity, corresponding to the edges of the DP gap, are marked by vertical black dashed lines. One can see that for the sample with large gap (panels a,b) the photoemission signal strongly depends on the polarization of LR. The most important observation is a pronounced modification of the intensity of the upper and lower DC states at opposite circular polarizations. For positive circular polarization, the intensity of the upper DC is enhanced. In contrast, for negative circular polarization an enhancement of the lower DC intensity is observed. Redistribution between the upper and lower DC states under circular polarization of LR is seen for both 9 and 35 K, while for the latter the intensity of the DC states is reduced. Panels a3,b3 and a4,b4 show this redistribution in details. In panels a3,b3 the circular dichroism (CD) ARPES dispersion maps are shown, which were derived by subtracting the spectra measured at opposite circular LR polarizations. Figure 5 (a4 and b4, upper panels) compare the EDCs measured at opposite circular polarizations in the region close to the DC gap. The lower panels demonstrate the corresponding CD signal obtained by subtraction of the EDCs presented in upper panels with normalization on their sum.

For the case of large gap at the DP at temperature below $T_{\mathrm{N}}$ (panels a3,a4), the CD-signal demonstrates a pronounced inversion between the upper and lower DC states. The change of the sign of CD signal correlates to some extent with the change of the spin polarization of the gapped DC state (see Ref. ^39^). To confirm such a correlation, the spectra are presented in the region including also the Te-derived exchange-split states at the edge of CB located in the energy region 0.14–0.22 eV (see for comparison Ref. ^23^). We have to note the presence of spin-polarized Mn-derived states weight in this energy region (see Fig. 2), which may contribute to CD signal. Thus, the CD signal sign inversion is not pronounced for CB states, that can be explained by the opposite spin-polarization of the Mn- and the Te-derived exchange-split states.

Above $T_{\mathrm{N}}$, when the long-range magnetic ordering is destroyed, the energy splitting between the edge CB states collapses, and no sign inversion is observed for these states in the CD signal (panel b4). At the same time, the sign inversion in the CD signal between the upper and lower DC states remains visible above $T_{\mathrm{N}}$. The same inversion can be distinguished in the ARPES dispersion map (panel b3). This may indicate the preservation of a magnetic origin of the gap above $T_{\mathrm{N}}$, possibly due to short-range order effects. The persistence of the gap above $T_{\mathrm{N}}$ had been explained by presence of strongly anisotropic spin fluctuations in the wide temperature range, see Ref. ^7^. While the exact mechanism of spin fluctuations requires further studies, we would like to note here the following points. Owing to the strong out-of-plane anisotropy it is highly expectable that Mn magnetic moments prefer out-of-plane direction even above $T_{\mathrm{N}}$. Taking into account relatively large defects concentration (from 3 to 17.5% of ${\hbox {Mn}}_{\mathrm {Bi}}$ and ${\hbox {Bi}}_{\mathrm {Mn}}$ antisite defects as well as Mn vacancies^40–42^) one can assume that Mn atoms in Bi layer also possess out-of-plane moments. These magnetic moments may induce the chiral spin texture in the surrounding media (similar to Ref. ^36^) that can affect the magnetic state of the crystal. On the other hand, competing magnetic interactions in ${\hbox {MnBi}}_2 {\hbox {Te}}_4$ can induce a number of various magnetic phases, including skyrmion ones (see Ref. ^43^). We speculate that the formation of the skyrmion-like spin textures can be generated at elevated temperatures in the Mn-Te bilayers inside SLs, like as it is in heterostructures MnTe/TI^44^.

Figure 5c1,d1 and c2,d2, show the ARPES dispersion maps and the corresponding EDCs for the sample with reduced gap, measured at temperatures below and above $T_{\mathrm{N}}$, respectively. First of all, it can be clearly seen in Fig. 5c2,d2 that the gap width does not depend on polarization of LR both below and above $T_{\mathrm{N}}$. It is interesting that both CD ARPES dispersion map (panel c3) and corresponding EDC (panel c4) measured for the Te- derived CB edge states below $T_{\mathrm{N}}$ demonstrate the pronounced inversion of the CD signal. At the same time, the inversion of the CD sign for upper and lower DC states is practically not observed. Whereas, some redistribution in the CD-signal between the upper and lower DC states can be distinguished in the CD ARPES dispersion maps. We associate this observation with a reduced effective magnetic moment developed for this kind of sample in the area of the topological DC state localization (see below). On the other hand, the exchange splitting of the CB states collapses above $T_{\mathrm{N}}$ (panel d4), that testifies rather to a developed FM magnetic coupling in the probing surface-contributed area. It means that despite the FM magnetic ordering probed by the exchange-split CB states, the DC states are affected by a significantly reduced effective out-of-plane magnetic moment. On the contrary, according to the theoretical modelling performed in Ref. ^14^ the “gapless”-like DC can arise in ${\hbox {MnBi}}_2 {\hbox {Te}}_4$ due to the surface magnetic reconstruction resulting in formation of a zero out-of-plane magnetic moment in the Mn-layer inside the surface SL. This may be due to (1) formation of the intralayer 2D AFM coupling (instead the 2D FM one), (2) in-plane arrangement of the magnetic moments in the surface SL and (3) formation of the surface spin-disordered paramagnetic-like layer. However, the presented experimental CD measurements (with spin-resolved data) likely demonstrate maintaining of the surface FM ordering. An alternative explanation of the gapless-like dispersion in such a case can be a possibility of topological surface state weight redistribution and gap decrease owing to the van-der-Waals space enlargement (see below).

### XMCD with varied applied out-of-plane magnetic field

**Figure 6:**
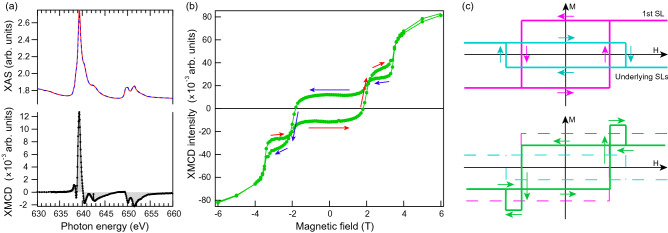
(**a**, upper panel) X-ray Absorption Spectra (XAS), with blue and red curves corresponding to the opposite circular polarizations of SR and (**a**, bottom panel) XMCD spectrum, i.e. the difference of the two XAS. The measurements have been made in the total electron yield mode at Mn $L_{2,3}$ edge (630–660 eV) at 15 K in zero magnetic field. (**b**) XMCD amplitudes at the Mn $L_{2,3}$ edge plotted as a function of applied out-of-plane magnetic field (linear background is subtracted). Arrows show direction of the curve moving from − 6 to 6 T (red) and back (blue). (**c**) Schematic presentation of contributions from topmost SL (magenta) and underlying SLs with effective opposite magnetization (cyan) to the XMCD signal as well as the resulting curve (green).

To study the surface magnetic ordering, we have carried out measurements of X-ray Magnetic Circular Dichroism (XMCD) which are more surface sensitive in comparison to SQUID measurements. XMCD signal is proportional to the sample magnetization either intrinsic or induced under applied magnetic field. Figure 6a shows the X-ray Absorption Spectra (XAS) and XMCD signal. One can see pronounce XMCD signal of Mn $L_{2,3}$ even in zero magnetic field. Thus, these results may indicate an FM magnetic ordering.

In order to study this peculiar result, we measured dependence of system magnetization (amplitude of XMCD signal) on applied magnetic field M(H), see Fig. 6b. Presented M(H) curve demonstrates a pronounced hysteresis loop also with FM-like behavior in an applied magnetic field below 2 T (above − 2 T). It differs from the M(H) curve, measured by bulk-sensitive SQUID method, which demonstrates well-known AFM character without hysteresis loops between a field of − 3.5 and 3.5 T (see Fig. 2e in Ref. ^7^). Moreover, an unusual deviation of FM-like M(H) curve is observed in the region between 2 and 3.5 T where it demonstrates reversed direction to the “main” loop. Appearance of these additional hysteresis loops can be observed in case of A-type AFM (as in ${\hbox {MnBi}}_2 {\hbox {Te}}_4$) due to following reasons. The XMCD method is surface sensitive so the signal rapidly decreases with a depth and is mainly provided by the contribution of the several top SLs. In case of A-type AFM each SL has FM ordering with opposite orientation to its neighbors. Therefore, topmost SL delivers FM behavior with maximum contribution in the signal which is schematically shown by magenta M(H) curve in Fig. 6c. Underlying SLs almost compensate each other though give some residual XMCD signal due to their inequivalent depth which can be presented as M(H) curve (cyan) with opposite direction. Besides the amplitudes, M(H) curves for the topmost SL and underlying SLs might show different magnetic coercivity. This can be caused by change in surrounding on the surface or/and possible structural relaxations. As seen from bottom part of panel (c) variation of coercivity is crucial for appearance of additional hysteresis loops. Resulting modeled XMCD signal (magnetization) qualitatively reproduce experimental observation in panel (b).

Thus, our magnetic measurements indicate an out-of-plane FM ordering with an XMCD signal coming mostly from the topmost SL. At the same time, XMCD measurements can be consistent with the A-type AFM ordering inherent to ${\hbox {MnBi}}_2 {\hbox {Te}}_4$. Also, our results demonstrate that in several cases a consideration of the magnetization contribution from the second and following SLs can be important. As we show in the following, their effect on the DP gap might be dramatic when the topological surface state partially relocates to the second SL due to the increase of the vdW spacing.

### Structural effect on surface electronic structure of $\mathbf{MnBi }_2 \mathbf{Te }_4$

**Figure 7:**
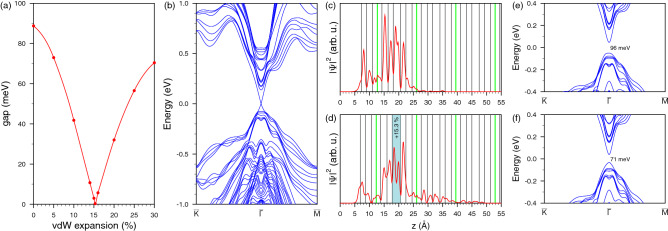
Dependence of the electronic structure of $\mathbf{MnBi }_2 \mathbf{Te }_4$ on structure alteration. (**a**) The gap in the Dirac state as function of the first van der Waals spacing expansion; (**b**) Electronic structure for the slab with the 1-st vdW spacing detached by 15.3%; Spatial charge distribution of the Dirac state at equilibrium structure (**c**) and for vdW spacing expanded by 15.3% (**d**); Surface electronic spectrum of ${\hbox {MnBi}}_2 {\hbox {Te}}_4$ with modified interlayer distances in the outer SL only (**e**) and the outer SL and the 1-st vdW spacing (**f**) as taken from Ref. ^40^.

The alteration of the topological surface state localization is possible due to structure modification caused by natural unavoidable defects. It was revealed that ${\hbox {MnBi}}_2 {\hbox {Te}}_4$ usually contains from 3 to 17.5% of ${\hbox {Mn}}_{\mathrm{Bi}}$ and ${\hbox {Bi}}_{\mathrm{Mn}}$ antisite defects as well as Mn vacancies^40–42^. Due to the fact that Mn and Bi atoms have noticeably different size it is evident that presence of a number of ${\hbox {Bi}}_{\mathrm{Mn}}$ defects in the Mn layer should expand average interlayer Te-Mn-Te distances in the middle of SL, and vice versa, the presence of ${\hbox {Mn}}_{\mathrm{Bi}}$ in the Bi layers should lead to a decrease in the Bi-Te average interlayer distances. Indeed, the structural parameters presented in Refs. ^40, 42^ show that Mn-Te and outer Te-Bi interlayer spacings are respectively by 3–3.5% expanded and contracted as compared to our calculated equilibrium interlayer distances whereas the second, Bi-Te, distance differs only within one percent from the theoretical value. At the same time these data for defect containing samples also show that vdW spacing between neighboring SLs is of $\sim$ 8–10% larger with respect to the calculated ${\hbox {MnBi}}_2 {\hbox {Te}}_4$ structure. Another structural effect earlier discussed^25^ for TIs with weak van der Waals coupling between building multilayered blocks is a broadening of vdW spacing near the surface caused by the mechanical cleavage or exfoliation implied for the surface preparation for ARPES and STM experiments. For ab-initio simulation of the structural effects we apply an approach that previously showed its efficiency in explaining the experimentally observed features in layered TIs spectra and which is based on consideration of changes in interlayer spacings within or between building blocks of TI^45–47^. In our simulation we restrict the structural changes only in the surface SL and in the first vdW gap, since it is known that the topological surface state is almost completely localized in this area. First we consider the effect of vdW spacing expansion on the surface electronic structure. As previously shown for layered TIs the gradual detachment of the outer block leads to progressive relocation of the topological state to the deeper QL^45^. For ${\hbox {MnBi}}_2 {\hbox {Te}}_4$ we have considered the vdW spacing expansion up to 30%, first, in increments of 5% and then, to find a minimum DP gap, with smaller steps. As one can see in Fig. 7a, the detachment of the outermost SL leads to rapid decrease in the DC gap and at experimentally determined values of expansion of $\sim$ 8–10% it is twice as smaller than in the equilibrium structure. An intriguing finding is that at 15.3% expansion of the first vdW spacing the topological surface state of ${\hbox {MnBi}}_2 {\hbox {Te}}_4$ AFMTI becomes gapless. The spectrum of the ${\hbox {MnBi}}_2 {\hbox {Te}}_4$ slab with outermost vdW spacing expanded by 15.3% (corresponding to 0.38 Å) is presented in Fig. 7b. With a further increase in the outermost vdW spacing, the DC gap rapidly increases. The strong dependence of the DC gap on the vdW spacing variation stems from the change in spatial localization of the topological surface state. Like in non-magnetic TIs, being initially localized mainly in the surface SL (Fig. 7c) where it is affected by magnetization from single Mn layer, it relocates inward and, as a result, begins to experience the magnetization provided by Mn atoms of the second SL which are characterized by opposite orientation of the magnetic moment. At certain expansion (which we identified is of 15.3%) the influence of opposite magnetic moments compensate each other (Fig. 7d). With further increase in the outermost vdW spacing the weight of the DC state in outer SL decreases even more and the influence of the Mn layer of the second SL becomes dominant.

To study the effect of the structural changes produced by antisite defects within SL we extracted the interlayer distances from the structural data presented by Zeugner et al.^40^. The spectrum of the ${\hbox {MnBi}}_2 {\hbox {Te}}_4$ slab with modified in accordance with the experimental interlayer distances outermost SL is shown in Fig. 7e. It demonstrates the DC gap (96 meV) a bit wider than that in the equilibrium structure (88 meV). After a series of calculations with a variation of the SL interlayer distances we found that the gap can be smaller than that in ideal ${\hbox {MnBi}}_2 {\hbox {Te}}_4$ due to reduction of the second, Bi-Te, spacing, but again not by much (at moderate variations). Finally, introducing additionally experimental vdW spacing into the structure, which is by 8% larger than theoretical spacing, we expectedly got the gap reduction (71 meV, see Fig. 7f). Thus, considering two structural effects, intrablock effect, provided by antisite defects within the SL block, and interblock vdW spacing expansion, provided by both antisite defects and mechanical cleavage distortions, we revealed that the former one does not affect much the DC gap, whereas the expansion of the vdW spacing can lead to wide variation in the gap width: from 88 meV in the ideal structure to zero. Hence the DC gap should depend strongly on a sample quality and surface preparation accuracy. The gap may even slightly exceed the theoretical value for the ideal structure due to antisite defects inside the SL.

## Conclusion

We have shown that both a large ($60 - 70~ \hbox {meV}$) and significantly reduced ($<20~ \hbox {meV}$) gap at the DP can be clearly distinguished using laser ARPES in the measured dispersion maps for different kinds of samples (or different surface areas) of the intrinsic AFM topological insulator ${\hbox {MnBi}}_2 {\hbox {Te}}_4$. The first value is close to the predicted from theoretical calculation, while the second one is close to the “gapless”-like dispersion assumed for the samples with significantly rearranged surface structural ordering. In both cases the gap at the DP remains open above $T_{\mathrm{N}}$, when the long-range magnetic ordering is destroyed, being only slightly decreased in magnitude. This conclusion is made on a basis of precise analysis of the gap size as a function of temperature across the AFM transition. In the ARPES dispersion maps, the magnetic transition manifests itself in a continuous decrease of the intensity of the DC gap state with temperature below $T_{\mathrm{N}}$, while above it the intensity of the DC state is almost constant.

The measured spin-resolved ARPES dispersions demonstrate a helical in-plane spin structure with inversion of the spin for the DC states with opposite momenta. The out-of-plane spin structure exhibits an inversion of the spin polarization between the upper and lower parts of DC. The sign of the spin polarization oscillates with photon energy due to the PE final state effect. These features of the in-plane and out-of-plane spin distributions are observed both below and above $T_{\mathrm{N}}$. We assume that the DP gap can remain open above $T_{\mathrm{N}}$ due to an emerging short-range magnetic field generated by chiral-like spin fluctuations.

A detailed analysis of results of the CD ARPES and XMCD measurements with varied magnetic field for the samples with “large” and “reduced gap” confirms the surface out-of-plane FM ordering. However, for the topological DC state the CD ARPES signal demonstrates the sign inversion for the “large gap” samples, while for the samples with the “reduced gap” it is not distinguished. By means of ab-initio simulations we have shown that the size of the gap at the DP can be strongly affected by structural surface relaxation via the vdW spacing modulation. This effect leads to inward shift of the DC state localization where it becomes influenced by Mn atoms of both first and second SLs, bearing opposite magnetic moments. It is followed by significant variation of the DP gap size in a wide range from 88 meV in case of the ideal structure and practically to zero even in the case of fairly moderate value of vdW spacing expansion of 0.38 Å (15.3%).

Our results demonstrate complex behavior of electronic and spin structure in ${\hbox {MnBi}}_2 {\hbox {Te}}_4$ which can be highly affected by structural disturbance and surface relaxation processes. Deeper understanding and further investigations of these effects may open the way to tune the mass of the Dirac fermions and, hence, to control the axion electrodynamics in intrinsic magnetic topological insulator.

## Methods

The measurements of the ARPES dispersion maps were carried at the $\mu$-Laser ARPES system at HiSOR (Hiroshima, Japan) with improved angle and energy resolution and a high space resolution of the laser beam (spot diameter around $5~ \upmu \hbox {m}$) using a Scienta R4000 analyzer with an incidence angle of the LR of $50^\circ$ relative to the surface normal^31^. For the experiment we used photons ($h\nu =6.3~\hbox {eV}$) of different polarizations (p-, s- and opposite circular ones).

The spin-resolved ARPES experiments were carried out using (1) s- polarized LR ($h\nu =7~\hbox {eV}$)^48^ at the ISSP, University of Tokyo and (2) SR with various photon energies at BL-9B endstation of HiSOR (Hiroshima, Japan).

The XMCD/XAS experiments were performed at the twin helical undulator beamline BL23SU of SPring-8^49^ in Japan.

High-quality ${\hbox {MnBi}}_2 {\hbox {Te}}_4$ single crystals were grown using the vertical Bridgman method at the Azerbaijan State Oil and Industry University and characterized by X-ray diffraction^13^ at the Institute of Physics of Azerbaijan National Academy of Science.

Part of the ARPES experiments were also carried out in the resource center “Physical methods of surface investigation” (PMSI) at the Research park of Saint Petersburg State University.

Clean surfaces of the samples were obtained by a cleavage in ultrahigh vacuum. The base pressure during all photoemission experiments was better that $1 \times 10^{-10}~ \hbox {mbar}$.

Ab-initio electronic structure calculations were carried out within the density functional theory using the projector augmented-wave (PAW) method^50, 51^ as implemented in the VASP code^52, 53^. The exchange-correlation energy was treated using the generalized gradient approximation^54^. The Hamiltonian contained scalar relativistic corrections and the Spin-orbit interaction was included in all types of calculations. In order to describe the van der Waals interactions we made use of the DFT-D3^55^ approach. The Mn 3*d*-states were treated employing the $\hbox {GGA}{+}U$ approach^56^ within the Dudarev scheme^57^. The $U_\text {eff}=U-J$ value for the Mn 3*d*-states was chosen to be equal to $5.34\,\mathrm {eV}$, as in previous works on ${\hbox {MnBi}}_2 {\hbox {Te}}_4$^7, 8, 58–61^.

## Data Availability

The authors declare that the data supporting the findings of this study are available within the paper.
